# Proteomics and transcriptomics reveal molecular subtypes and biomarkers of advanced cutaneous T-cell lymphoma

**DOI:** 10.3389/fonc.2026.1849456

**Published:** 2026-07-15

**Authors:** Shan Zhang, Zhengguang Guo, Zhaorui Liu, Zhiyu Pang, Feng Qi, Haidan Sun, Wei Sun, Jie Liu

**Affiliations:** 1 Department of Dermatology, State Key Laboratory of Complex Severe and Rare Diseases, Peking Union Medical College Hospital, Chinese Academy of Medical Science and Peking Union Medical College, Beijing, China; 2 Proteomics Research Center, Core Facility of Instrument, Institute of Basic Medical Sciences & School of Basic Medicine, Chinese Academy of Medical Sciences & Peking Union Medical College, Beijing, China

**Keywords:** biomarker, cutaneous T-cell lymphoma, laser capture microdissection, molecular subtypes, mycosis fungoides, proteomics, transcriptomics

## Abstract

**Background:**

Cutaneous T-cell lymphomas (CTCL) are a group of non-Hodgkin T-cell lymphomas, with mycosis fungoides and Sézary syndrome being the most common subtypes. Advanced-stage CTCL are typically aggressive, exhibiting interpatient heterogeneity in treatment response and prognosis. The underlying pathogenesis remains incompletely elucidated, posing challenges for the selection of appropriate therapies.

**Methods:**

We obtained tumor cell-enriched regions of 33 advanced CTCL samples from 31 patients using laser capture microdissection, followed by integrated proteomic and transcriptomic profiling. Selected biomarkers were further validated via immunohistochemistry.

**Results:**

We identified three molecular subtypes of advanced CTCL, which exhibited significant differences in clinical phenotypes, signature proteins, and pathways. These subtypes were designated as intracellular signaling subtype, metabolic subtype, and extracellular matrix remodeling subtype, respectively. Within intracellular signaling subtype, the PI3K-AKT-mTOR pathway was characteristically upregulated, and we found the expression level of phospho-AKT was associated with response to PI3Kδ inhibitor therapy. Comparative proteomic analysis of patients with varying treatment responsiveness and disease progression identified CTSB, GSTO1, and WDFY4 as potential biomarkers for predicting treatment responsiveness, and GOLGA1 and STIP1 as potential biomarkers for progression prediction.

**Conclusion:**

This study explored a potential molecular subtyping framework for advanced-stage CTCL associated with different clinical phenotypes. Our findings provided preliminary evidence suggesting that certain biomarkers may be associated with treatment response to PI3K inhibitors. Additionally, we screened and preliminarily identified candidate biomarkers that may be associated with treatment responsiveness and progression risk, which may assist clinicians in the management of advanced-stage CTCL. Notably, these molecular differences may also correlate with clinical characteristics and require validation in larger cohorts.

## 1 Background

Cutaneous T-cell lymphoma (CTCL) is a group of rare primary cutaneous non-Hodgkin lymphomas that exhibit distinct molecular biological characteristics, clinical manifestations, and treatment responses compared to nodal lymphomas. Mycosis fungoides (MF) and Sézary syndrome (SS) are the most common subtypes of CTCL, accounting for approximately 65% of all CTCL cases (1). Early-stage MF typically presents with skin erythema and plaques, progressing slowly, and most patients achieve disease control with standardized treatment, such as regular phototherapy, topical agents, or systemic immunomodulatory therapy, resulting in a favorable prognosis. In contrast, advanced-stage CTCL may manifest as skin tumors or erythroderma, with potential involvement of lymph nodes, blood, or other organs. Therapeutic options for advanced disease are diverse, including interferon, oral methotrexate, oral retinoids, histone deacetylase inhibitors, brentuximab vedotin, mogamulizumab, PI3K inhibitors, and chemotherapy (2). However, treatment responses are highly variable among patients, with some exhibiting aggressive disease progression despite multiple lines of therapy.

Advanced-stage CTCL poses significant challenges in clinical management. Previous transcriptomic and proteomic studies have sought to identify drivers of CTCL progression by characterizing molecular alterations in advanced disease. In the field of proteomic research, comparative studies on protein expression differences between MF and inflammatory dermatoses skin samples have identified DYNC1I2, CD14, COL18A1, and CRABP2 as potential biomarkers for early diagnosis of MF (3). Gan et al. (4) conducted a proteomic study comparing early- and late-stage MF lesions, revealing differentially expressed proteins were involved in biological processes such as DNA replication initiation, nucleosome assembly, and cellular response to external stimulus, as well as pathways including bacterial invasion of epithelial cells, focal adhesion, Toll-like receptor signaling, and β-alanine metabolism. Using transcriptomic sequencing, Xiao et al. (5) observed significant transcriptomic alterations during late-stage disease progression, with changes in pathways such as MEK/ERK, AKT-mTOR, and T cell activation. Additionally, transcriptomic studies have revealed that PD-1 is a hallmark of exhausted CTCL, and loss of PD-1 is associated with a worse prognosis (6). Häyrinen et al. (7) compared the transcriptomic profiles of early-stage MF patients with different responses to skin−directed therapy and found that the subgroup with poor prognosis exhibited activation of molecules related to keratinocytes, basal cell populations, and melanocytes. Furthermore, some molecules and pathways associated with disease progression have been identified in advanced-stage MF using single-cell transcriptomics. A study comparing single-cell transcriptomic profiles between long-standing patches and newly developed plaques/tumors in advanced-stage MF patients found downregulated expression of CXCR4, CD69, and HSPA1A in progressed lesions (8). Song et al. (9) analyzed single-cell transcriptomes of 16 large cell-transformed CTCL skin samples and demonstrated that tumorigenesis and progression were associated with oxidative phosphorylation, cellular plasticity, upregulation of MYC and E2F activity, and downregulation of MHC I.

Advanced-stage CTCL exhibits significant heterogeneity in clinical manifestations, therapeutic responses, and prognosis. Patients may present with infiltrative flat tumors, exophytic fungating tumors, or erythroderma, yet the molecular drivers of these distinct phenotypes remain largely undefined. Clinically, while the overall prognosis is poor, patient outcomes following intervention are highly variable. A subset of individuals may achieve remission with agents such as interferon-α or oral methotrexate, while others require more aggressive targeted therapies or chemotherapy, and some patients continue to progress despite multiple lines of treatment. Risk stratification based on treatment responsiveness and progression risk is critical for determining therapeutic intensity and improving patient outcomes. However, the mechanisms underlying the heterogeneity in treatment response and prognosis remain unclear, and current proteomic and transcriptomic findings have yet to fully delineate the molecular basis of clinical diversity in advanced CTCL. In this study, we utilized laser capture microdissection to isolate tumor-cell-enriched regions from patient samples, followed by proteomics and transcriptome sequencing, which revealed three molecular subtypes of advanced CTCL and analyzed their molecular characteristics. Within one of the subtypes, patient-level validation further identified p-AKT as a potential biomarker for predicting treatment response to PI3Kδ inhibitors. Furthermore, we categorized patients into different subgroups based on their treatment responsiveness and prognosis. By comparing proteomic profiles between these groups, we explored potential biomarkers that may help predict treatment responsiveness and progression risk, thereby contributing to the future development of stratified management strategies for advanced CTCL.

## 2 Materials and methods

### 2.1 Patient recruitment

A total of 31 patients with advanced-stage CTCL were enrolled from the Department of Dermatology, Peking Union Medical Collage Hospital in the period from 2019 to 2023. Inclusion criteria were as follow: (1) confirmed diagnosis of MF or SS based on the criteria of the International Society for Cutaneous Lymphomas and the cutaneous lymphoma task force of the European Organization of Research and Treatment of Cancer (10); (2) TNMB stage IIB-IVB; and (3) availability of punch or surgical biopsies of skin tissue specimens from typical lesional sites.

We collected the onset age, gender, stage, serum LDH level, skin lesion type, therapy, and the time from diagnosis to progression of each patient. All participants gave written informed consent in accordance with the Declaration of Helsinki for specimen collection and analysis under the study protocol approved by the Peking Union Medical College Hospital Ethics Committee (JS-3409).

### 2.2 Laser-capture microdissection

Fresh-frozen tissue samples from all 33 samples were processed for subsequent proteomic analysis. For each sample, fresh-frozen tissues were embedded in OCT regent and stored at -80 °C to generate tissue blocks. A 5-µm-thick section was prepared on a metal-frame polyethylene terephthalate (PET) membrane slide (MMI, Prod. No. 50103, Germany). Before laser microdissection, the membrane slide was treated with H_2_O for 30 seconds to remove OCT and rehydrate the tissue, followed by 70% ethanol for 15 seconds to dehydrate and fix the sections. Targeted tumor cells enriched regions were isolated via pressure catapulting using a 20× objective under brightfield optics on an MMI CellCut system (MMI, Germany). Approximately 100–500 cells, representing an area of 0.5-1 mm² per sample, were collected (**Supplementary Figure 1**). The isolated cells were transferred into 0.5 mL transparent caps (MMI, Prod. No. 50204, Germany) and resuspended in extraction buffer, followed by the preparation protocol detailed below.

### 2.3 Protein sample preparation

A 20 µL extraction solution (10 mM Tris, 0.8 mM EDTA, 0.2% Zwittergent 3-16) was added to each collection cap containing the dissected cell samples. The samples were then denatured using 20 mM dithiothreitol (DTT) at 60°C for 2 hours with sonication. Following denaturation, alkylation was performed with 55 mM iodoacetamide (IAM) for 45 minutes at room temperature in the dark. The samples were subsequently loaded onto a 10 kDa molecular weight cutoff filter membrane and centrifuged at 14,000 × g for 20 minutes. The membrane was washed five times with 20 mM Tris buffer and then subjected to tryptic digestion (enzyme-to-protein ratio of 1:50) at 37 °C overnight. Finally, the resulting peptides were collected by centrifugation.

### 2.4 Liquid chromatography with tandem mass spectrometry

Peptide samples were analyzed using a timsTOF Pro 2 mass spectrometer (Bruker Daltonics) coupled to the U3000 (Thermo Fisher) liquid chromatograph in data-independent acquisition (DIA) mode. Digested peptides (4μL) were separated on a monolithic silica C18 column (75 μm × 15 cm, Omitech, Beijing) using a 20-minute linear gradient from 5% to 30% mobile phase B (0.1% formic acid in acetonitrile) at a flow rate of 0.5 μL/min. For DIA acquisition in dia-PASEF mode, we employed 62 variable isolation windows optimized based on precursor m/z distribution of a pooled sample to equalize ion counts across windows. The full scan range was set from 350 to 1280 m/z. Electrospray ionization (ESI) used a 1700 V capillary voltage. TIMS inverse reduced mobility (1/K0) data were collected over a range of 0.71–1.36 1/K0.

To assess the reproducibility of the MS system, a quality control (QC) mixture prepared from pooled urine samples was analyzed before and after each batch of samples, as well as after every 20 injections.

### 2.5 Data processing

The raw DIA data were processed using Spectronaut Pulsar 17.1 (Biognosys) with default settings. Briefly, the data were searched against the human SwissProt database (Homo sapiens, 20,358 entries, 2019_05 release), allowing up to two missed tryptic cleavages. Cysteine carbamidomethylation was set as a fixed modification, while methionine oxidation, lysine deamination, and carbamylation (+43 Da) were included as variable modifications. Dynamic iRT was used for retention time prediction. Interference correction at the MS1 level was enabled. Peptide intensities were calculated by summing MS1 fragment ion peak areas, and protein intensities were derived from the summed peptide intensities. Protein inference was performed using the ID picker algorithm, with results filtered at 1% FDR (Q-value < 0.01). For quality control, a pooled sample was analyzed to monitor system stability. Proteins with >50% missing values in QC samples were excluded, while remaining missing values were imputed using the K-nearest neighbors method (for proteins with ≤50% missing values) or global minimum value (for proteins with >50% missing values). The imputed data were subjected to median normalization (**Supplementary Figure 2**).

### 2.6 RNA seq and data processing

RNA-seq was performed by Chi-biotech cooperation (Shenzhen, China). Total RNA was extracted from tissues or cells, quantified by Nanodrop, and assessed for integrity using an Agilent 4200 TapeStation. Poly(A) mRNA was enriched using Oligo(dT) magnetic beads, fragmented, and reverse-transcribed into double-stranded cDNA, followed by end repair, A-tailing, adapter ligation, size selection (200-300 bp), and PCR amplification to generate the sequencing library. The qualified libraries were sequenced on an Illumina NovaSeq 6000 platform with a PE150 read mode. Read counts were normalized using the median of ratios normalization method in DESeq2 to eliminate the variations in sequencing depth and RNA composition in the samples (11). Normalized counts were logarithmically transformed with base 2. A total of 17,091 transcripts with read counts > 1 in at least 50% of the samples were retained for downstream analysis.

### 2.7 Statistical analyses

We used non-negative matrix factorization (NMF) consensus clustering for molecular typing. For the NMF method, the standard “brunet” option was selected, and 50 iterations were carried out. The number of clusters k was set as 2 to 8, and the average contour width of the common member matrix was determined through the R package “NMF.” The optimal cluster number was selected based on the cophenetic correlation coefficient, the average silhouette width, and inspection of the consensus matrix. The cophenetic coefficient peaked at k=3 and then markedly decreased, while the average silhouette width at k=3 was 0.9, indicating excellent cluster quality. Furthermore, the consensus matrix at k=3 showed three distinct, uniformly dark red diagonal blocks with sharp boundaries and clean off-diagonal regions. Accordingly, k=3 was determined as the optimal number of clusters (**Supplementary Figure 3**).

In the analysis of clinical information, we used one-way analysis of variance (ANOVA) to compare the mean age at onset across three clusters, and the student t-test to compare the mean age and mean LDH levels between two subgroups. Fisher’s exact test was used to compare categorical variables, such as gender, disease stage, skin lesion types, proteomic subtype, and treatment efficacy, between different subgroups (SPSS 22.0, IBM Corporation, US). For the TNMB stage among subgroups with different treatment responsiveness and progression risks, the Cochran−Armitage test was applied to evaluate the association between disease stage and clinical characteristics using R (v4.6.0, R Core Team, 2026), the DescTools package (v0.99.60, Andri Signorell, 2025). The statistical results are presented in **Tables 1**, **2**, and **Supplementary Tables 6**, **7**. Survival was estimated by the Kaplan-Meier method, and any differences in survival were evaluated with a log-rank test (GraphPad Prism 10, GraphPad Software, US).

**Table 1:** Clinical data of enrolled patients.

Characteristic	Cluster1 (N = 15)	Cluster2 (N = 8)	Cluster3 (N = 8)	Total	P value
Gender					0.64
Male	8	4	6	18	
Female	7	4	2	13	
Diagnosis					0.31
MF	14	8	6	28	
SS	1	0	2	3	
Stage					0.015*
IIB	9	6	1	16	
IIIA	0	2	4	6	
IIIB	1	0	0	1	
IVA	3	0	2	5	
IVB	2	0	1	3	
Skin lesion type					0.001*
Fungating tumor	7	0	0	7	
Flat tumor	6	6	1	13	
Erythroderma	2	2	7	11	
Large cell transformation					0.36
Yes	4	2	0	6	
No	11	6	8	25	
Treatment					1.0
Targeted therapy or chemotherapy	9	5	4	18	
Immunomodulatory therapy	6	2	3	11	
Not available	0	1	1	2	
Outcome					0.67
Progression	6	4	2	12	
Stable or response	7	2	4	13	
Not available	2	2	2	6	
Serum lactic dehydrogenase					1.0
Normal	8	3	4	15	
Elevated	7	4	4	15	
Not available	0	1	0	1	

*p value<0.05.

**Table 2:** Baseline characteristics and treatment response of patients.

Characteristic	Response (N = 3)	Non-response (N = 4)	Total	P value
Age, median (range), y	50 (36-68)	40 (30-44)	43 (30-68)	0.20
Gender				0.49
Male	2	1	3	
Female	1	3	4	
Clinical stage				0.26
IIB	3	1	4	
III	0	0	0	
IVA	0	1	2	
IVB	0	2	2	
Skin lesion type				0.11
Fungating tumor	3	1	4	
Flat tumor	0	3	3	
Erythroderma	0	0	0	
Liperlisib dose (per day)				0.43
40mg	1	0	1	
80mg	2	4	6	
Treatment Efficacy				0.09
Complete response	1	0	1	
Partial response	2	0	2	
Stable disease	0	1	1	
Disease progression	0	3	3	

To identify cluster signature proteins/genes, firstly, comparisons between multiple clusters were conducted using one-way ANOVA; cluster differences resulting in p-values of less than 0.05 were considered to be statistically significant. Then, a cluster signature protein/gene was defined as a protein/gene with an abundance level in one cluster of at least 1.5-fold of the other two clusters.

To identify differential proteins in different tumor-type, different treatment responsiveness, and different prognoses, a student t-test was conducted, p-value < 0.05 and fold change > 1.5 were considered to be statistically significant. The ROC curve was visualized, and the AUC was calculated using MedCalc.

### 2.8 Downstream bioinformatics analysis

Functional analysis of the proteins of interest was performed using two complementary approaches. First, Gene Ontology (GO) enrichment analysis was conducted through the “Wu Kong” platform (https://wkomics.omicsolution.com/wkomics/wkold/), with all identified human proteins serving as the background reference. Second, we utilized Ingenuity Pathway Analysis (IPA) software (Ingenuity Systems, Mountain View, CA) to evaluate signature proteins and differentially expressed proteins. The IPA analysis examined disease associations, molecular functions, and canonical pathways. In GO and IPA enrichment analysis, the p-value is calculated using the right-tailed Fisher’s exact test without adjustments, p-value < 0.05 as statistical significance. In IPA disease and biofunction analysis, the Z-score was calculated to predict activation states. Z score > 0, activated; Z score < 0, inhibited.

### 2.9 Immunohistochemistry

An immunohistochemical staining procedure was performed according to the instructions provided. Paraffin sections (4 μm) containing human advanced CTCL skin tissue specimens were deparaffinized, hydrated, and heated to 95–100 °C for 4 min to induce antigen retrieval. After inactivating endogenous peroxidase activity, rabbit anti-human STIP1 monoclonal antibody (1: 500 dilutions, #ab129106; Abcam); rabbit anti-human GOLGA1 polyclonal antibody (1: 150 dilutions, #ab84340; Abcam); rabbit anti-human CTSB monoclonal antibody (1: 50 dilutions, #ab125067; Abcam); rabbit anti-human GSTO1 monoclonal antibody (1: 200 dilutions, #ab129106; Abcam); rabbit anti-humanWDFY4 polyclonal antibody (1 : 100 dilution, #17558-1-AP; Proteintech); rabbit anti-human AKT1 monoclonal antibody (1 : 1000 dilution, #80816-1-RR; Proteintech); mouse anti-human phospho-AKT monoclonal antibody (1 : 200 dilution, #166444-1-Ig; Proteintech) were used to perform IHC staining. Antibody diluents, chromogenic agents, and other related reagents were purchased from Leica Microsystems.

Quantitative assessment of immunohistochemical staining intensity was performed using the average optical density (AOD) value (Image J, National Institutes of Health). AOD was treated as a continuous quantitative measure, with higher AOD values indicating stronger DAB staining. DAB-positive signal regions were segmented using the IHC Toolbox of ImageJ software, with an AOD threshold range of 0.174-2.708 to exclude non−specific background staining. The AOD value was calculated according to the formula: AOD = Integrated Optical Density (IOD)/Area. Comparisons of mean AOD values between the two groups were conducted using the t-test, and a p-value < 0.05 was considered statistically significant (GraphPad Prism 10, GraphPad Software, US).

## 3 Results

### 3.1 Patient characteristics

A total of 31 patients with advanced-stage CTCL were enrolled, including 18 males and 13 females. The mean age at disease onset was 45.8 years. According to the TNMB staging system (2), all patients were classified as stage IIB-IVB. Based on literature and clinical practice, we categorized the skin lesions of advanced CTCL patients into three types: ①Flat tumor-type lesions: manifested as flat, infiltrative tumors. ②Fungating tumor-type lesions: characterized by hemispherical nodules or tumors (12). ③Erythrodermic-type lesions: presented as diffuse erythema affecting more than 80% of the body surface area (**Supplementary Figure 4A**). Among the 31 patients included in this study, 7 presented with the fungating tumor-type lesions, 13 exhibited the flat tumor-type lesions, and 11 were the erythrodermic type (**Table 1**, **Supplementary Table 1**).

### 3.2 Molecular subtyping of advanced CTCL based on proteomics

**Figure 1:**
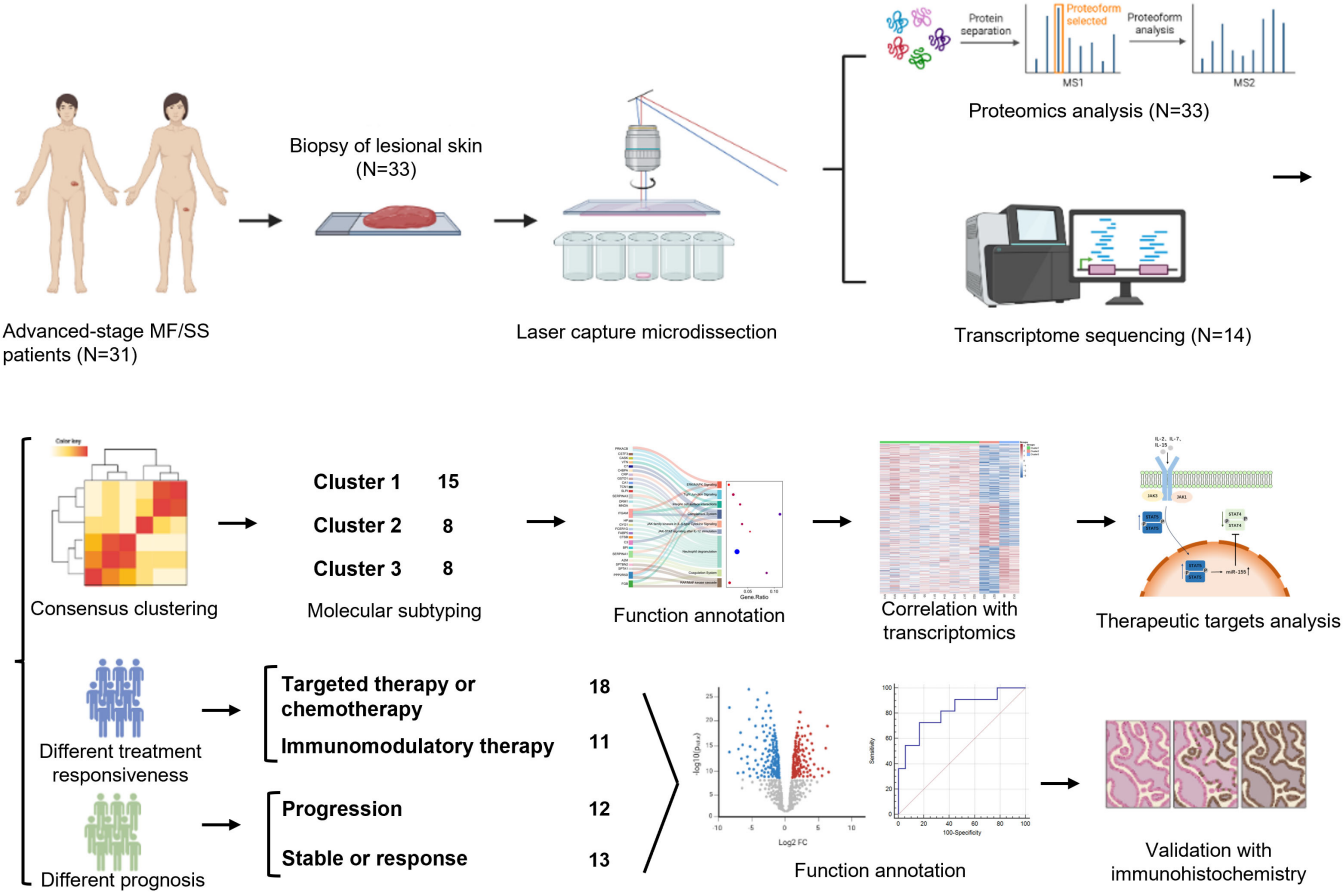
Diagram of workflow of this study. 33 frozen skin tissue sections were obtained from 31 patients with advanced CTCL. Tumor cell-enriched regions were obtained using laser capture microdissection and subjected to proteomic analysis, and 14 samples were also analyzed by transcriptome sequencing. We performed molecular subtyping of all samples and characterized subtype-specific molecular profiles. Additionally, we analyzed patients with differential treatment responsiveness and prognostic outcomes to identify predictive biomarkers.

We collected 33 frozen skin tissue sections from 31 patients with advanced CTCL (2 patients provided two skin lesion samples from different locations). Tumor cell-enriched regions were obtained using laser capture microdissection and subjected to data-independent acquisition (DIA) proteomic analysis (**Figure 1**). Proteomic analysis identified a total of 5,407 proteins (average 4314 and 5150 proteins in samples and QCs). After excluding proteins with missing values exceeding 50% in quality control and samples, 5,194 proteins were quantified. Unsupervised principal component analysis (PCA) analysis indicated that all QC samples were clustered together, and correlation analysis suggested the average correlation coefficient of QC samples was 1.00, demonstrating the stability of the MS analysis (**Supplementary Figures 3A–C**).

For the RNA-seq data, 17 LCM samples by RNA-seq were analyzed, and 14 samples passed the quality control (RIN ≥ 6, 28S/18S ≥ 0.7, gene mapping ≥ 40%) and were used for further analysis. An average of 25575 transcripts was identified in each sample. After excluding transcripts with missing values exceeding 50% in samples, 20,340 transcripts were quantified (**Supplementary Figures 3A–D**).

**Figure 2:**
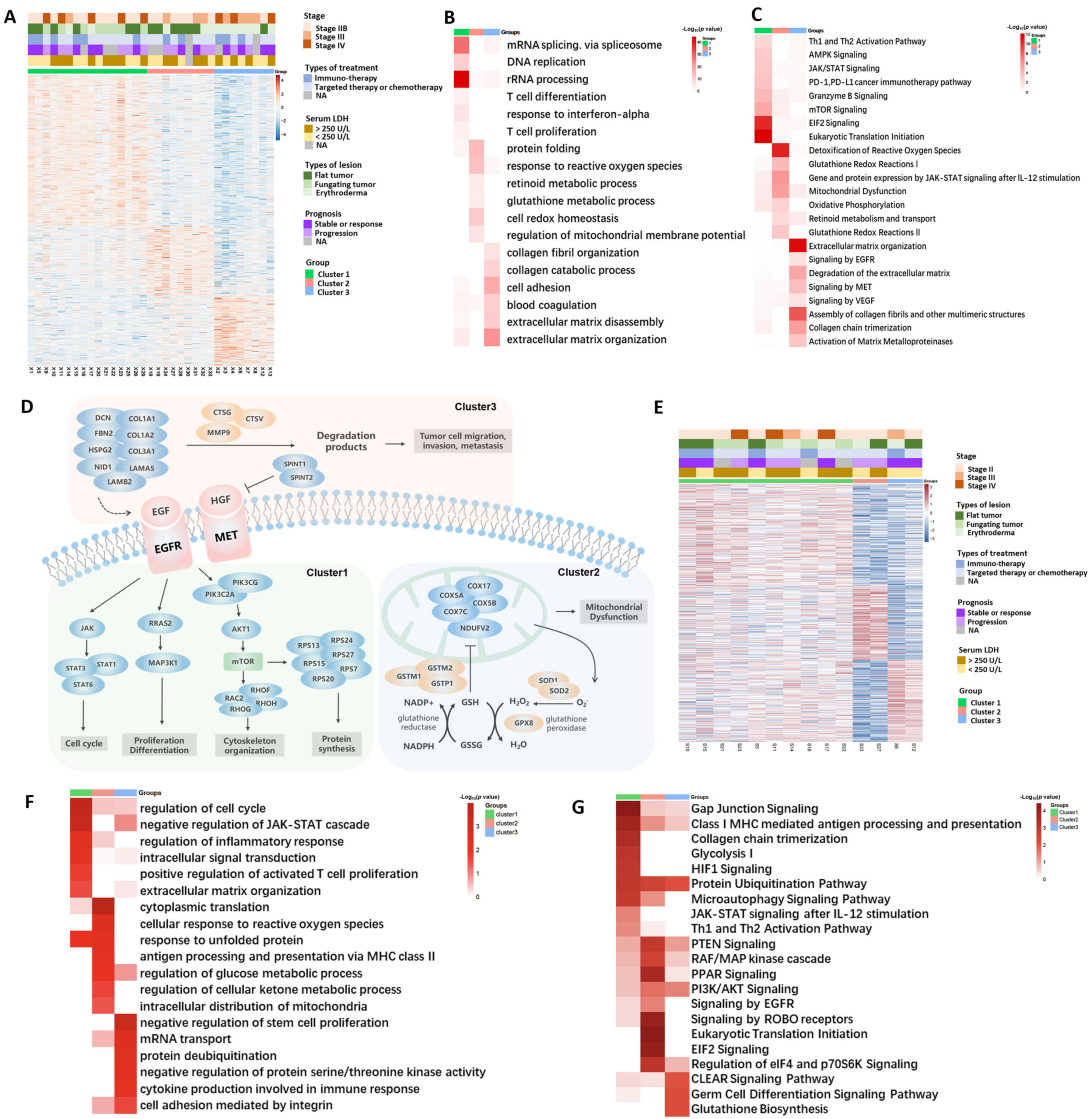
Molecular typing based on proteome and association analysis with transcriptome data. **(A)** Proteomic subtypes and clinical information of 33 advanced-stage CTCL skin specimens. **(B)** Heatmap displaying the biological processes associated with the characteristic proteins in each subtype as determined by GO enrichment analysis. **(C)** Heatmap illustrating the key pathways involved in the three subtypes. **(D)** Key pathways involved in different subtypes of advanced-stage CTCL. The molecules indicated by the blue and yellow circles are proteins and enzymes detected by proteomics. **(E)** Signature genes of three subtypes identified one-way ANOVA. **(F)** Heatmap displaying the biological processes associated with the signature genes in each subtype as determined by GO enrichment analysis. **(G)** Heatmap showing the key pathways involved in the three subtypes according to IPA of signature genes.

Based on the expression profiles of these proteins, NMF consensus clustering was performed, dividing all samples into three subgroups. The consensus matrix, cophenetic plot, and silhouette plot (**Supplementary Figure 3**) were used to group 33 samples into 3 clusters (detailed data in Methods section, **Figure 2A**, **Table 1**). Cluster 1 included 16 samples (two of which were from the same patient). Cluster 2 comprised 9 samples (two of which were from the same patient). Cluster 3 consisted of 8 samples.

The mean ages at disease onset for the three clusters were 41.5, 46.5, and 53.3 years, respectively, with no statistically significant differences (p > 0.05). Fisher’s exact test was used to compare the distribution of patients in different stages across the clusters, revealing statistically significant differences (p = 0.015). Among all patients, 25 had available prognostic information. We analyzed the time from diagnosis to disease progression in these patients. Kaplan-Meier survival analysis showed no statistically significant differences in progression-free survival among the three clusters (p = 0.94) (**Supplementary Figure 4B**). The patient’s specific TNMB stage, treatment, and follow-up data are shown in **Supplementary Table 1**.

We analyzed the association between the lesion types and proteomic profiles and found a correlation between lesion types and proteomic subtypes. Cluster 1 primarily consisted of fungating tumor-type (n = 7, 7/15) and flat tumor-type lesions (n = 6, 6/15), with all fungating tumor-type lesions exclusively found in this cluster. Cluster 2 included 6 flat tumor-type (6/8) and 2 erythrodermic-type lesions (2/8). Cluster 3 was predominantly composed of erythrodermic-type lesions (n = 7, 7/8). The distribution of lesion types across the three clusters showed statistically significant differences (p = 0.001).

In summary, proteomic-based molecular subtyping revealed that advanced CTCL can be classified into three distinct subtypes, with significant differences in staging and clinical phenotypes among the subtypes.

### 3.3 The three subtypes exhibit distinct characteristic proteins and key pathways

Using analysis of variance, we identified proteins upregulated in each subtype compared to the other two subtypes (p-value < 0.05 and fold change > 1.5). A total of 771, 210, and 480 characteristic proteins were identified for the three subtypes, respectively (**Figure 2A**, **Supplementary Table 2**). To elucidate the functional characteristics of these subtype-specific proteins, we performed Gene Ontology (GO) enrichment analysis and Ingenuity Pathway Analysis (IPA).

GO enrichment analysis revealed that the characteristic proteins in Cluster 1 were primarily involved in the regulation of gene expression, including rRNA processing, RNA splicing, DNA replication, and T cell differentiation (**Figure 2B**). IPA further highlighted upregulated pathways such as the EIF2 signaling, mTOR signaling, granzyme B signaling and JAK/STAT signaling pathway (**Figure 2C**), which are closely associated with intracellular signal transduction, cell growth, proliferation, and development. Previous studies have shown that the development and progression of CTCL are driven by complex intracellular signaling networks, including the JAK/STAT and EIF2 pathways (13–15), consistent with our findings. Based on these protein expression characteristics, we designated Cluster 1 as the “intracellular signaling subtype.”

GO enrichment analysis indicated that the characteristic proteins in Cluster 2 were mainly involved in protein folding, response to reactive oxygen species (ROS), cell redox homeostasis, regulation of mitochondrial membrane potential, and retinoid metabolism (**Figure 2B**). IPA results demonstrated enriched pathways related to mitochondrial dysfunction, glutathione redox reactions, and detoxification of ROS, all of which are closely linked to redox and metabolic processes (**Figure 2C**). Therefore, Cluster 2 was designated as the “metabolic subtype.”

GO enrichment analysis of Cluster 3 characteristic proteins revealed their involvement in biological processes such as extracellular matrix (ECM) organization, cell adhesion, ECM disassembly, blood coagulation, and collagen catabolic process (**Figure 2B**). IPA results further highlighted pathways associated with ECM remodeling, including ECM organization and degradation, collagen biosynthesis and modifying enzymes, and activation of matrix metalloproteinases (MMPs). Additionally, pathways related to cell surface receptor-mediated signaling, such as the signaling by MET, signaling by platelet-derived growth factor, and epidermal growth factor receptor, were also enriched (**Figure 2C**). ECM remodeling has been implicated in the progression and invasiveness of CTCL. Previous studies have shown upregulated expression of MMP-2 and MMP-9 in MF (16), aligning with our finding that Cluster 3 characteristic proteins are involved in MMP activation. Based on these features, Cluster 3 was designated as the “extracellular matrix remodeling subtype.” The enrichment analysis results are shown in **Supplementary Table 3**.

In summary, our findings demonstrate significant differences in the functional characteristics and enriched pathways of the characteristic proteins among the three subtypes. The clinical phenotypes of the subtypes differ in lesion types and stages, which are known to be associated with the biological behavior and disease progression risk. Our findings highlight the distinct gene expression profiles of advanced CTCL patients behind the clinical phenotypes, revealing molecular-level heterogeneity and providing new insights into the pathogenesis of different subtypes (**Figure 2D**).

### 3.4 Correlation analysis of transcriptomic and proteomic results

Transcriptomic sequencing was performed on 14 samples simultaneously. Based on the proteomic classification results, 10 samples belonged to the “intracellular signaling type,” while the “metabolic type” and the “extracellular matrix type” each included 2 samples (**Supplementary Table 4**). Comparisons between three clusters were conducted using one-way ANOVA, and a total of 241, 172, and 192 characteristic genes were identified for the three subtypes, respectively (**Figure 2E**). GO enrichment analysis and IPA were performed on the characteristic genes of each subtype.

The results showed that characteristic genes of the intracellular signaling subtype are involved in biological processes such as cell cycle regulation, regulation of the JAK/STAT pathway, intracellular signal transduction, and activated T cell proliferation (**Figure 2F**). IPA revealed pathways including gap junction signaling, class I MHC−mediated antigen processing and presentation, collagen chain trimerization, and glycolysis (**Figure 2G**). Similar to the proteomic findings, transcriptomic analysis also indicated that the characteristic genes of this subtype are associated with intracellular signal transduction processes such as the JAK/STAT pathway, but additionally featured pathways related to immune responses and energy metabolism.

The characteristic genes of the metabolic subtype are involved in cytoplasmic translation, cellular response to reactive oxygen species, regulation of glucose metabolism, and ketone metabolism (**Figure 2F**). IPA showed pathways including PPAR signaling, eukaryotic translation initiation, and EIF2 signaling (**Figure 2G**). The metabolism−related biological functions identified in the transcriptomic analysis, such as cellular response to reactive oxygen species and glucose metabolism, were consistent with the proteomic findings, while transcriptomics also revealed functional features related to the regulation of gene expression and tumor-related pathways.

The characteristic genes of the extracellular matrix subtype are involved in biological processes such as negative regulation of stem cell proliferation, mRNA transport, protein deubiquitination, and cell adhesion (**Figure 2F**), implicating pathways including the CLEAR signaling pathway and glutathione biosynthesis (**Figure 2G**). Compared with the proteomic results, the transcriptomic features of this subtype did not show obvious extracellular matrix remodeling functions; instead, the signature genes were primarily associated with protein modification and regulation of transcription and translation.

Taken together, these analyses demonstrate varying degrees of correlation between the transcriptomic and proteomic data across the different subtypes, and each subtype also exhibits transcriptome−specific molecular functions.

### 3.5 Heterogeneity analysis of the “intracellular signaling subtype” patients

**Figure 3:**
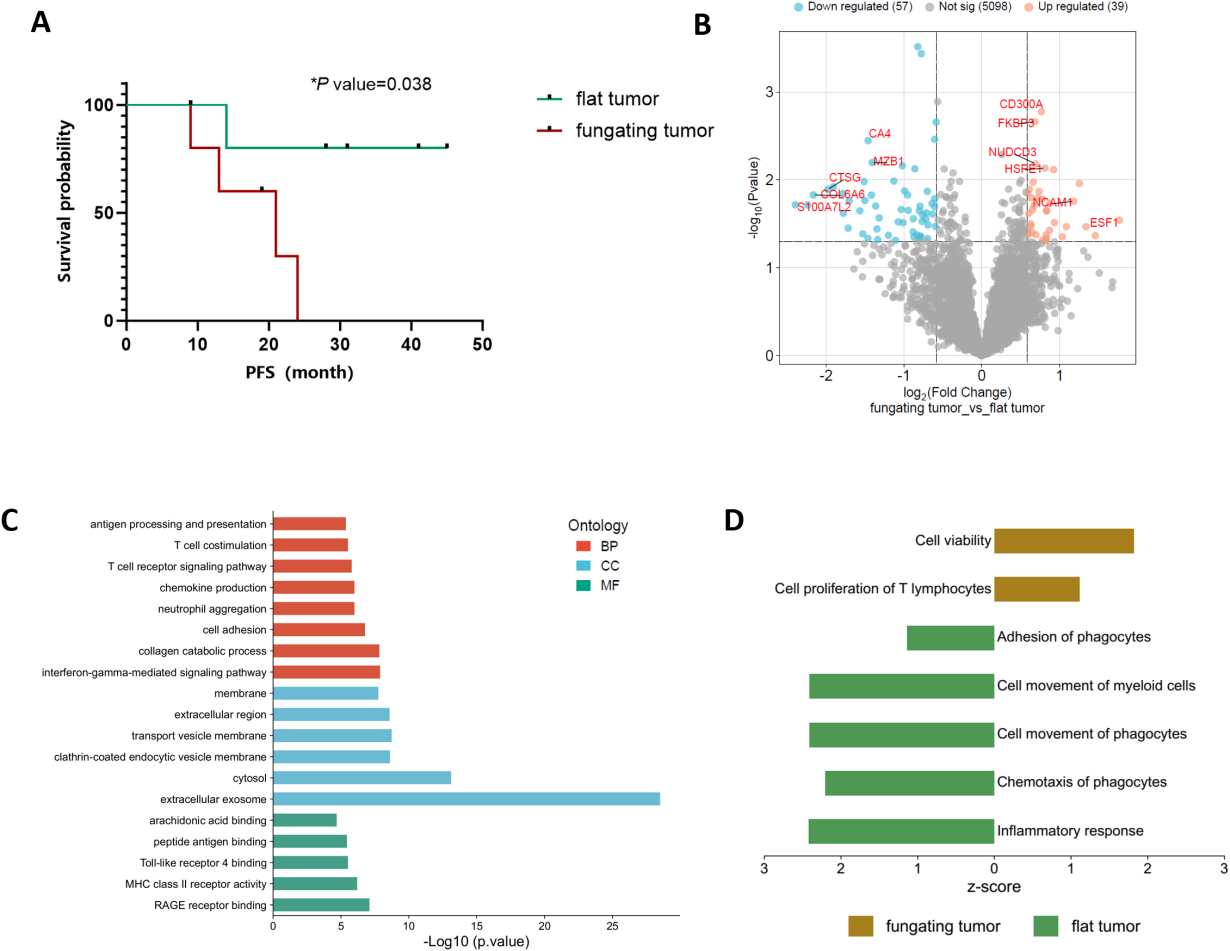
Heterogeneity analysis of the “intracellular signaling subtype”. **(A)** Kaplan-Meier curves showing a statistically significant difference in time to disease progression between patients with flat tumor-type (n=6) and fungating tumor-type (n=5) lesions (Log-rank test, p = 0.038). **(B)** Volcano plot displaying DEPs between patients with flat tumor-type and tumor-type lesions. **(C)** GO enrichment analysis results of DEPs between flat tumor-type and tumor-type lesions. **(D)** IPA biofunction analysis of DEPs between flat tumor-type and tumor-type lesions (Z-score > 0, upward trend; Z-score < 0, downward trend). DEP, differentially expressed protein; GO, Gene Ontology; BP, Biological Process; MF, Molecular Function; CC, Cellular Component.

Among patients classified as the “intracellular signaling subtype”, we observed that the majority exhibited clinical phenotypes of flat tumor-type lesions (n = 6, 6/15) and fungating tumor-type lesions (n = 7, 7/15), with a similar number of patients in both groups. To investigate whether there were differences in prognosis between these two groups, we analyzed 11 patients with available prognostic information. The results showed a statistically significant difference in disease progression between the two groups (p = 0.038), with patients presenting flat tumor-type lesions having a better prognosis (**Figure 3A**, **Supplementary Table 5**).

We further explored differentially expressed proteins (DEPs) between the two groups and identified 96 DEPs (**Figure 3B**). GO enrichment analysis of the DEPs revealed their involvement in biological processes such as interferon-γ-mediated signaling, collagen catabolic process, cell adhesion, and neutrophil aggregation (**Figure 3C**). Among these, CD300A and FKBP3 were highly expressed in the fungating tumor-type lesion group, while CA4 and MBZ1 were upregulated in the flat tumor-type lesion group (**Figure 3B**). In studies of hematologic malignancies, high expression of CD300A and FKBP3 has been associated with poor prognosis (17, 18). Conversely, MBZ1, which plays a critical role in humoral immunity, has been linked to tumor progression in colorectal cancer when downregulated (19). These findings align with our observation that patients with fungating tumor-type lesions had a worse prognosis.

IPA biofunction analysis indicated that, compared to the flat tumor-type group, the fungating tumor-type group exhibited upregulated biological functions related to T cell proliferation of T lymphocytes and cell viability, while processes such as inflammatory response, cell movement of phagocytes, and myeloid cells, chemotaxis of phagocytes and adhesion of phagocytes were downregulated (**Figure 3D**). These results suggest that patients with fungating tumor-type lesions have more active tumor cell proliferation and weakened anti-tumor immunity, consistent with the clinically observed higher aggressiveness and poorer prognosis in this group.

### 3.6 Biomarker for PI3K inhibitor therapy efficacy

Analysis of “intracellular signaling subtype” revealed that its characteristic proteins are involved in multiple intracellular signal transduction pathways, including PI3K-AKT-mTOR and JAK-STAT signaling. Dysregulation of these pathways has been implicated in the pathogenesis and progression of cancers, underlying potential targets for anticancer drugs, suggesting that patients of this subtype may benefit from the targeted therapies. Notably, several antitumor drugs targeting the PI3K-AKT-mTOR pathway have been approved for clinical use. The PI3Kδ isoform plays a critical role in lymphocyte signal transduction and activation, rendering PI3Kδ inhibitors particularly promising therapeutic agents for the treatment of lymphomas (20).

Our previous clinical trial demonstrated that the selective PI3Kδ inhibitor linperlisib, combined with the histone deacetylase inhibitor chidamide, achieved an objective response rate of 59.1% in the relapsed or refractory CTCL patients (21). However, in clinical practice, this treatment is currently applied on a trial basis only to patients with relapsed or refractory advanced-stage CTCL, and there is a lack of validated biomarkers to predict treatment efficacy.

**Figure 4:**
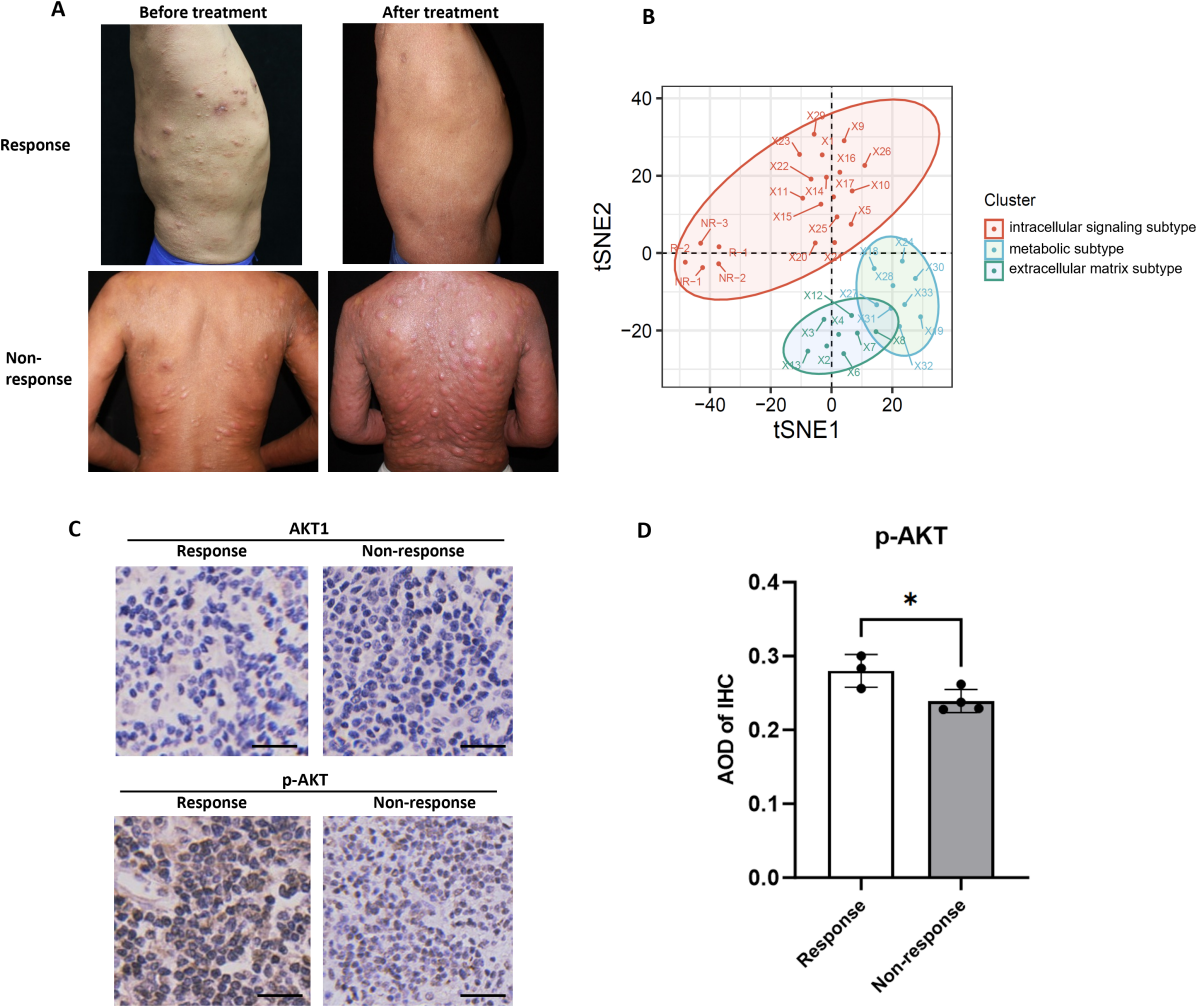
Different response to PI3K inhibitor treatment. **(A)** Clinical images before and after linperlisib and chidamide treatment of response group and non-response group. **(B)** t-SNE plot showing the seven patients receiving PI3K inhibitor therapy (sample X20, X25 and five newly analyzed samples) belonging to “intracellular signaling subtype.” **(C)** Immunohistochemical staining of AKT1 and p-AKT (scale bar=50μm). **(D)** T-test showed a statistically significant difference of AOD of p-AKT staining, and the response group had higher positivity (p=0.035). R, response; NR, non-response; AOD, average optical density.

To explore the association between treatment response and molecular subtype, we selected a cohort including seven patients receiving linperlisib and chidamide treatment for at least one treatment cycle. Baseline characteristics and treatment response data are summarized in **Table 2**. The cohort included 3 male and 4 female individuals, with a median age of 43 years (range 30-68). According to the TNMB staging system, all the patients were classified as advanced stage, with 4 individuals of IIB stage, and each 2 individuals belonging to the IVA and IVB stages. As for the skin lesion type, four patients had fungating tumor-type and three had flat tumor-type lesions. All patients received chidamide at a fixed dose of 20 mg twice weekly. Linperlisib was administered at a dose of 40 mg once daily in one patient and 80 mg once daily in the others. Best of treatment response was assessed according to International Society for Cutaneous Lymphomas/United States Cutaneous Lymphoma Consortium/European Organization for Research and Treatment of Cancer criteria (22). Three patients with partial or complete response were classified as the response subgroup, and four patients with stable disease or disease progression as the non-response subgroup. Representative clinical images before and after treatment of each group were shown in **Figure 4A**.

We obtained pre-treatment frozen skin tissue sections and collected tumor cell-infiltrated regions via laser microdissection, followed by DIA proteomic analysis. Clustering analysis confirmed that all seven patients belonged to the “intracellular signaling subtype” (**Figure 4B**).

Mechanistically, PI3K activates AKT through phosphorylation, thereby regulating downstream signaling. To further identify whether the difference of activation level of the PI3K-AKT-mTOR pathway results in the different treatment response, we performed immunohistochemical (IHC) staining for AKT1 and phosphorylated AKT (p-AKT) on pre-treatment skin tissues from all seven patients. The staining results showed no significant difference in AKT1 expression between the two groups. However, p-AKT positivity was significantly higher in treatment-responsive patients, whereas tumor cells in non-responsive patients largely lacked p-AKT expression (**Figure 4C**, **Supplementary Figure 5**). IHC staining intensity was quantified using the average optical density (AOD). The threshold range (0.174–2.708) was used for image segmentation to exclude non−specific background staining (see Methods for details). Statistical analysis revealed a significant difference in the AOD of p-AKT staining between the two groups (p=0.035) (**Figure 4D**). The results indicate that patients who responded to linperlisib exhibited higher activation of the PI3K-AKT-mTOR pathway, rendering them more sensitive to PI3K inhibition.

In summary, proteomic profiling of seven patients treated with a PI3Kδ inhibitor and a histone deacetylase inhibitor confirmed that all belonged to the “intracellular signaling subtype.” Subsequent immunohistochemical analysis revealed elevated p-AKT expression in treatment-responsive patients, with no significant difference in AKT1 levels between the two groups. This suggests that the activation level of the PI3K-AKT-mTOR pathway may correlate with treatment response to PI3K inhibitors-based therapy, and p-AKT could represent a candidate biomarker associated with PI3Kδ inhibitor efficacy in patients of the “intracellular signaling subtype”, although this finding requires prospective validation.

### 3.7 Proteomic characteristics and biomarker identification in patients with different treatment responsiveness

Patients with advanced CTCL exhibit significant differences in treatment response. Some patients can achieve disease remission or long-term stabilization with immunomodulatory treatments such as interferon or oral methotrexate, while others require more aggressive targeted therapies or chemotherapy to control their disease, with some still experiencing disease progression despite multiple lines of treatment.

To identify molecular differences and potential biomarkers for predicting treatment responsiveness, we compared the proteomic profiles of patients with different levels of treatment responsiveness. Patients were categorized into two groups based on the intensity of treatment required: the “immunomodulatory therapy group” (n=11) and the “targeted therapy or chemotherapy group” (n=18). The “immunomodulatory therapy group” was defined as patients who achieved disease remission or stabilization with treatments such as interferon, retinoids, and methotrexate. The “immunomodulatory therapy group” consisted predominantly of male patients (9/11, 81.9%), whereas the “targeted therapy or chemotherapy group” had a roughly equal number of male and female patients. A higher proportion of patients with stage IVA−IVB disease was observed in the “targeted therapy or chemotherapy group” (7/18, 38.9%), but the Cochran−Armitage trend test showed no statistically significant difference (p = 0.12). The mean LDH level was higher in the “targeted therapy or chemotherapy group”, yet with considerable within−group variability, and the difference between the two groups did not reach statistical significance. Regarding lesion types, the “immunomodulatory therapy group” predominantly presented with flat tumor−type (6/11, 54.5%) and erythrodermic lesions (4/11, 36.4%), while in the “targeted therapy or chemotherapy group”, all three lesion types were equally represented, each with six patients. In terms of molecular subtyping, the “intracellular signaling subtype” was the most common in both groups (54.5% and 50%, respectively). Clinical information of involved patients is shown in the **Supplementary Table 6**.

**Figure 5:**
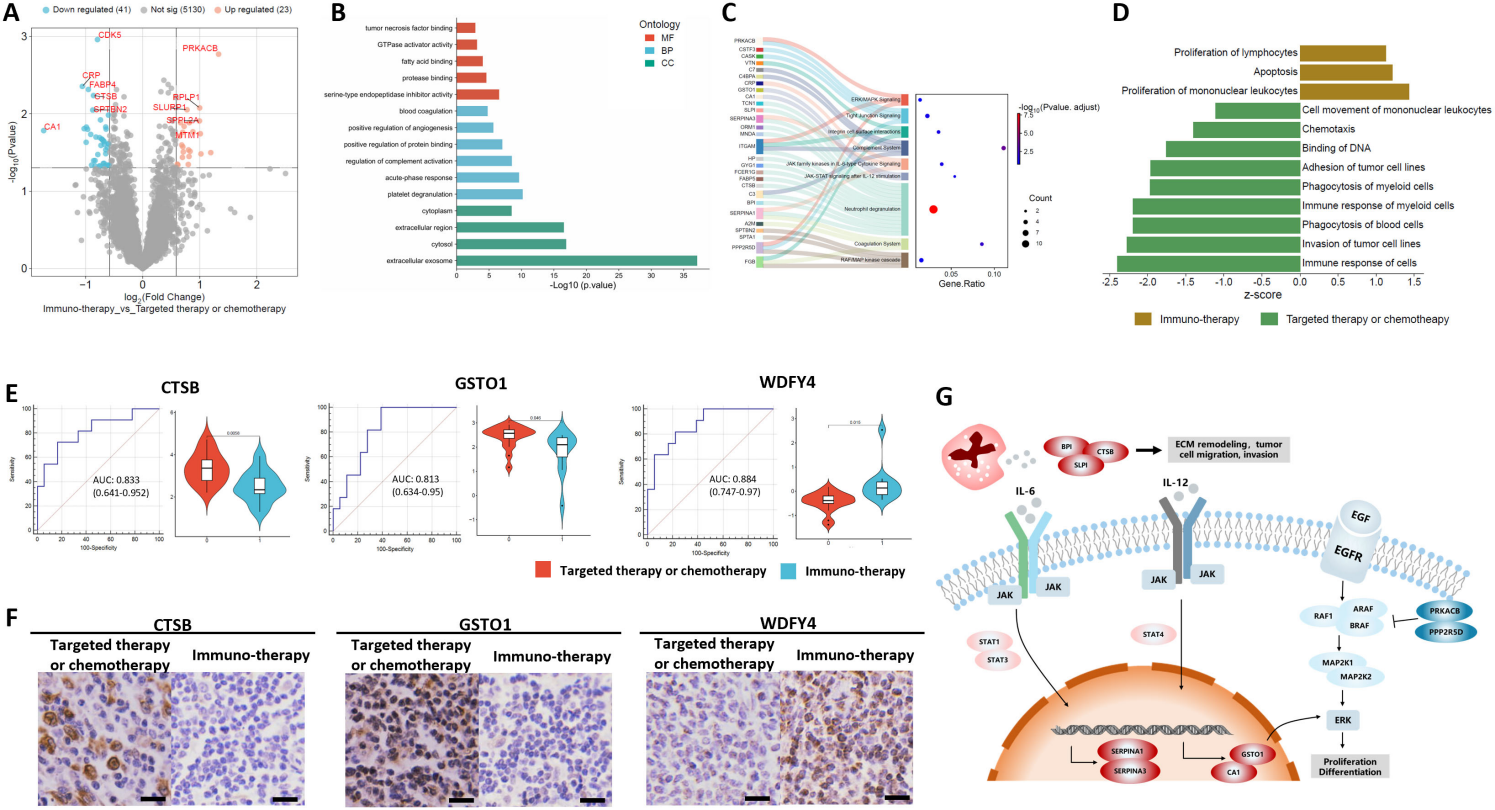
Differentially expressed protein analysis in patients with different treatment responsiveness. **(A)** Volcano plot displaying DEPs between the immunomodulatory therapy group (n=11) and the targeted therapy or chemotherapy group (n=18). **(B)** GO enrichment analysis results of DEPs between the “immunomodulatory therapy group” and the “targeted therapy or chemotherapy group”. **(C)** Enriched pathways of DEPs between the “immunomodulatory therapy group” and the “targeted therapy or chemotherapy group” based on IPA analysis. **(D)** IPA biofunction analysis of DEPs between the “immunomodulatory therapy group” and the “targeted therapy or chemotherapy group” (Z-score > 0, upward trend; Z-score < 0, downward trend). **(E)** ROC curves showing the performance of CTSB, GSTO1, and WDFY4 expression levels in distinguishing between the “immunomodulatory therapy group” and the “targeted therapy or chemotherapy group”. The violin plot showed the statistical difference between “immunomodulatory therapy group” (n=11) and the “targeted therapy or chemotherapy group” (n=18) using Student’s T-test, and the p-value were shown in the violin plot. **(F)** Immunohistochemical staining demonstrating significant differences in the positive staining rates of CTSB, GSTO1, and WDFY4 between the “immunomodulatory therapy group” and the “targeted therapy or chemotherapy group” (Scale bar=30μm). **(G)** Pathway Diagram of proteins associated with treatment responsiveness. The molecules indicated by the red circles are DEPs upregulated in the “targeted therapy or chemotherapy group” and dark blue circles are DEPs downregulated in the “targeted therapy or chemotherapy group”. DEP, differentially expressed protein; GO, Gene Ontology; IPA, Ingenuity Pathway Analysis; BP, Biological Process; MF, Molecular Function; CC, Cellular Component.

By comparing the proteomic results of the two groups, we identified a total of 64 DEPs. 23 proteins were downregulated in the “targeted therapy or chemotherapy group,” with the most significant differences observed for PRKACB, which mediates cAMP-dependent signaling (**Figure 5A**). Low PRKACB expression has been found to be associated with poor prognosis in colorectal cancer and non-small cell lung cancer (23, 24). GO enrichment analysis revealed that the DEPs were involved in biological processes such as platelet degranulation, acute-phase response, regulation of complement activation, and angiogenesis (**Figure 5B**). The enriched pathways for DEPs included neutrophil degranulation, RAF/MAP kinase pathway, JAK-STAT pathway, ERK/MAPK pathway, complement system, and integrin cell surface interactions (**Figure 5C**). IPA biofunction analysis indicated that, compared with patients requiring targeted therapy or chemotherapy, the “immunomodulatory therapy group” exhibited upregulation in biological functions such as proliferation of mononuclear leukocytes, apoptosis and proliferation of lymphocytes, while functions such as invasion of tumor cell lines, tumor cell adhesion, phagocytosis of blood cells, binding of DNA, cell movement of mononuclear leukocytes, and cell migration were downregulated (**Figure 5D**).

To identify potential biomarkers for predicting treatment responsiveness, we evaluate the ability to distinguish between the two groups of patients of the 64 DEPs by calculating the area under the Receiver Operating Characteristic (ROC) curve. According to the area under curve (AUC), protein abundance, and function, we selected the three most significantly differentially expressed proteins with tumor-relevant functions for further validation. Among these, two proteins were upregulated in the “targeted therapy or chemotherapy group” (CTSB and GSTO1) and one protein was downregulated in this group (WDFY4) (**Figure 5E**), all with an AUC greater than 0.8. We subsequently performed IHC staining on a new patient cohort, including 4 patients in the “immunomodulatory therapy group” and 6 in the “targeted therapy or chemotherapy group”, to validate the predictive efficacy of these three biomarkers. The staining results were analyzed using AOD values as the method described above, which showed significant differences in the staining positivity rates for these three biomarkers between the two groups, consistent with the proteomic findings (**Figure 5F**; **Supplementary Figures 6A–C**), suggesting that these proteins may serve as potential biomarkers for predicting treatment responsiveness. The possible mechanism for the difference in treatment responsiveness is shown in **Figure 5G**. In patients of “targeted therapy or chemotherapy group”, neutrophil degranulation leads to the release of proteins such as CSTB, which promotes extracellular matrix remodeling and tumor cell invasion. In addition, JAK/STAT pathway activation leads to the increased expression of proteins such as GSTO1, which promotes tumor cell survival by activating the ERK pathway.

In summary, by comparing the proteomic characteristics of advanced CTCL patients with different treatment responsiveness, we identified proteins and pathways associated with treatment responsiveness, and screened candidate biomarkers that may correlate with treatment responsiveness. Of note, the identified molecular changes may be associated with clinical phenotypes, as the patients with different treatment responsiveness differ in clinical characteristics, such as clinical stages and lesion types, which are linked to treatment response. These exploratory findings analyze the heterogeneity of treatment responsiveness at the molecular level and provide preliminary evidence that could potentially guide patient management.

### 3.8 Proteomic characteristics and biomarker identification in patients with different prognoses

Patients with advanced-stage CTCL generally have a poor prognosis, but disease aggressiveness varies among individuals. Previous studies have identified stage IV disease, age over 60 years, large cell transformation, and elevated serum LDH levels as independent risk factors for poor prognosis (25). Although clinical stage is known to correlate with progression risk, the molecular differences that underlie or correlate with these clinical distinctions remain incompletely characterized. Here, we analyzed the proteomic profiles of patients with different prognoses and aimed to investigate key prognosis-related proteins and their functions at the molecular level.

Among patients with available prognostic information, disease progression was defined by meeting any of the following criteria: ①progression in TNMB staging; ②an increase in the modified Severity-Weighted Assessment Tool (mSWAT) score by ≥25% from baseline, or in patients with complete or partial response, an increase of mSWAT score of greater than the sum of nadir plus 50% baseline score; ③patient death. A total of 12 patients were classified into the “progression group” (6 patients met criterion ①, one met criterion ②, and 5 met criterion ③), while 13 patients remained stable or achieved disease remission were included in the “stable or response group.”

The “progression group” included 5 male and 7 female patients, whereas the “stable or response group” comprised 9 male and 4 female patients. With respect to TNMB stage, stage IVA was the most prevalent in the “progression group” (4/12, 33.3%), while stage IIB predominated in the “stable or response group” (9/13, 69.2%), with a Cochran−Armitage test p−value of 0.06. Mean LDH levels were higher in the “progression group”, but the difference between the two groups did not reach statistical significance (p = 0.30). Lesion type distribution differed between groups: the “progression group” had roughly equal proportions of the three lesion types, whereas flat tumor−type lesions were the most frequent in the “stable or response group” (8/13, 61.5%). Regarding molecular subtypes, the “intracellular signaling subtype” was the most common in both the “progression group” (6/12, 50%) and the “stable or response group” (7/13, 53.8%). The “metabolic subtype” was the second most frequent in the “progression group” (4/12, 25%), while the “extracellular matrix remodeling subtype” was the second most frequent in the “stable or response group” (4/13, 30.8%). Clinical information for all included patients is provided in **Supplementary Table 7**.

**Figure 6:**
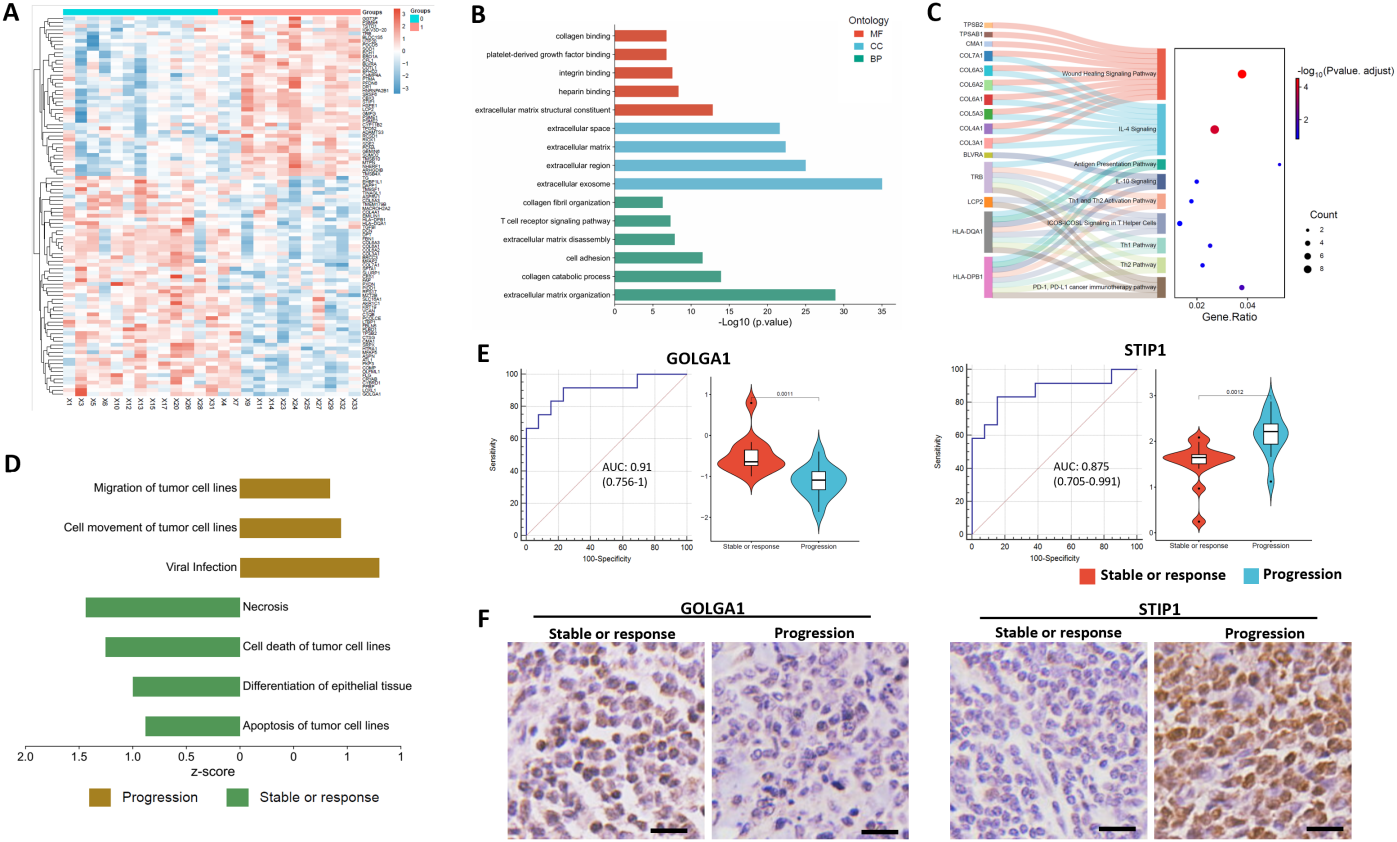
Differentially expressed protein analysis in patients with different prognoses. **(A)** Heatmap displaying DEPs between the progression group (n=12) and the stable or response group (n=13). **(B)** GO enrichment analysis results of DEPs between the “progression group” and the “stable or response group”. **(C)** Enriched pathways of DEPs between the “progression group” and the “stable or response group” based on IPA analysis. **(D)** IPA biofunction analysis of DEPs between the “progression group” and the “stable or response group” (Z-score > 0, upward trend; Z-score < 0, downward trend). **(E)** ROC curves showing the performance of GOLGA1 and STIP1 expression levels in predicting patients in the “progression group” and the “stable or response group”. The violin plot showed the statistical difference between “progression group” (n=12) and the “stable or response group” (n=13) using Student’s T-test, and the p-value were shown in the violin plot. **(F)** Immunohistochemical staining demonstrating significant differences in the positive staining rates of GOLGA1 and STIP1 between the “progression group” and the “stable or response group” (Scale bar=30μm). DEP, differentially expressed protein; GO, Gene Ontology; IPA, Ingenuity Pathway Analysis; BP, Biological Process; MF, Molecular Function; CC, Cellular Component.

Comparative proteomic analysis between the two groups identified 100 DEPs (**Figure 6A**). GO enrichment analysis revealed that the DEPs were involved in biological processes such as extracellular matrix organization, collagen catabolic processes, cell adhesion, and T-cell receptor (TCR) signaling pathway (**Figure 6B**). IPA indicated that these proteins were implicated in signaling pathways related to cellular stress and damage (e.g., injury repair pathways), immune response (e.g., ICOS-ICOSL signaling, IL-4 signaling, and Th1/Th2 pathways), and tumor-related pathways (e.g., PD-1/PD-L1 signaling) (**Figure 6C**).

IPA biofunction analysis demonstrated that patients in the “progression group” exhibited upregulation of function such as viral infection, cell movement of tumor cell lines, and migration of tumor cell lines, while apoptosis and cell death of tumor cell lines, differentiation of epithelial tissue, and necrosis were downregulated (**Figure 6D**). These findings suggest that disease aggressiveness in the progression group is primarily associated with reduced tumor cell apoptosis and enhanced migration and motility.

To identify prognostic biomarkers, we evaluated the ability of the 100 DEPs to distinguish between the “progression group” and the “stable or response group” using ROC curve analysis. Based on AUC values, protein abundance and function, we identified TMSB10, ECH1, GOLGA1, STIP1, and LTBP1, which had the greatest differences in expression and were functionally related to tumors, as potential prognostic biomarkers, ultimately selecting two candidates for further validation: GOLGA1, which was downregulated in the “progression group”, and STIP1, which was upregulated in the “progression group” (**Figure 6E**). The prognostic value of these biomarkers was further validated by IHC staining in a separate cohort of 4 patients in the “stable or response group” and 5 patients in the “progression group”. The AOD values for GOLGA1 and STIP1 were compared between the two groups as continuous variables, and the results demonstrated significant differences in the expression levels of these biomarkers between the two groups, consistent with the proteomic findings, indicating their potential utility as prognostic biomarkers (**Figure 6F**; **Supplementary Figures 6D, E**).

In conclusion, by comparing the proteomic characteristics of advanced-stage CTCL patients with different prognoses, we identified proteins and pathways associated with disease aggressiveness and potential prognostic biomarkers. Of note, these molecular differences are also associated with clinical phenotypes (e.g., stage and LDH) that are known to correlate with progression risk. These findings provide preliminary molecular insights into prognostic differences among patients and lay the foundation for future clinical translation.

## 4 Discussion

Here, we used laser capture microdissection to characterize the proteomic and transcriptomic characteristics of advanced-stage CTCL. By obtaining tumor cell-enriched regions from skin tissue sections, we ensured that the proteomic and transcriptomic results primarily reflected tumor cell characteristics, minimizing the influence of epidermal components and collagen fibers.

Systematically profiling the molecular landscape of tumors using omics technologies and subsequent precise classification and treatment based on molecular characteristics is a growing trend in cancer research (26, 27). Liu et al. (28) classified 13 cases of CTCL based on single-cell transcriptomic data into cytotoxic effector memory T cell and central memory T cell groups, and found that patients of the central memory T cell group exhibited more aggressive disease and poorer prognosis. This highlights the intrinsic heterogeneity of CTCL. However, large-scale proteomics-based subtyping studies for CTCL remain scarce.

In this study, using proteomic data from 33 advanced-stage CTCL skin tissue samples, we identified three molecular subtypes, each characterized by distinct molecular features associated with intracellular signal transduction, energy metabolism, and extracellular matrix remodeling. The “intracellular signaling subtype” was associated with pathways such as JAK/STAT, EIF2 signaling, PI3K-AKT-mTOR signaling, and NF-κB activation. Previous studies have shown that the development and progression of CTCL are linked to dysregulation of intracellular signaling pathways and transcription factor activation, including JAK/STAT, EIF2, PI3K-AKT, and NF-κB pathways (5, 29), consistent with our findings.

In terms of treatment, several target therapies for lymphomas aiming these pathways are currently in clinical use, such as the JAK1 inhibitor golidocitinib (30), PI3K inhibitors linperlisib and duvelisib. However, these novel therapies are effective only in a subset of patients, and no predictive biomarkers are currently available to guide treatment selection. Based on our previous clinical trial, we also investigated the therapeutic efficacy and biomarkers of linperlsib and chiadamide in a new cohort, and proteome analysis confirmed all seven patients of the “intracellular signaling subtype”. We investigated the expression of molecules associated with the PI3K-AKT-mTOR pathway across different treatment response subgroups using IHC staining. Our findings revealed a significant correlation between the activation level of this pathway and patient treatment responses. Specifically, the expression level of p-AKT showed a statistically significant difference between responders and non-responders. The result indicates that high p-AKT expression is predictive of a favorable treatment response, showing preliminary evidence of a potential association with response to PI3Kδ inhibitor-based therapy, although validation in larger cohorts is required. Future studies could further investigate the therapeutic efficacy and predictive markers of other agents in this patient subtype to guide clinical medication.

Additionally, multiple proteomic and transcriptomic studies have also revealed alterations in biological processes such as oxidative phosphorylation, oxidative stress response, cell adhesion, and extracellular matrix composition in CTCL (4, 13, 31), aligning with the molecular features observed in the other two subtypes. Recently, single-cell transcriptomic studies have shown that malignant T cells in CTCL exhibit oxidative phosphorylation metabolic signatures (9). Spatial transcriptomic studies have also indicated that CD30+ areas in MF-LCT tissues highly express genes related to extracellular matrix remodeling, further confirming that tumor cells are characterized by metabolic and extracellular remodeling signatures (32). Currently, for the characteristic pathways identified in these two subtypes, glutathione metabolism and activation of MMPs, anti-tumor drugs targeting glutathione S-transferases inhibitors and matrix metalloproteinase inhibitors are under development (33, 34), which are expected to provide targeted drug options for patients of “metabolic subtype” and “extracellular matrix remodeling subtype.”

Through correlation analysis of the proteomic classification results with clinical information, we found no statistically significant differences in prognosis among the three subtypes. This may be attributed to the small sample size and missing prognostic data for some patients. However, the subtyping results were associated with clinical phenotypes: patients in the “intracellular signaling subtype” primarily presented with fungating tumor-type and flat tumor-type lesions, the “metabolic subtype” mainly exhibited flat tumor-type lesions, and the “extracellular matrix remodeling subtype” predominantly showed erythrodermic-type lesions. These findings suggest that different clinical phenotypes not only reflect different disease stages but also indicate distinct underlying molecular mechanisms. Based on our clinical observations, patients with fungating tumor-type tend to have a higher tumor burden, more aggressive disease, and poorer prognosis. This was confirmed by the internal analysis of the “intracellular signaling subtype”, as patients with flat tumor-type lesions had a better prognosis than those with fungating tumor-type lesions. However, prognosis may be associated with factors such as genetic mutation status, necrosis, and overall tumor burden. Future studies with larger sample sizes are required to incorporate multidimensional data to explore the association between clinical phenotypes, proteomic features, and prognosis in larger patient cohorts.

There is currently no standard treatment for advanced-stage CTCL, and the heterogeneity of treatment responses among patients remains a major challenge in clinical practice. According to National Comprehensive Cancer Network (NCCN) guidelines, systemic therapies with fewer side effects are recommended as initial treatment, such as immunomodulatory treatments including interferon and low-dose methotrexate (2). A retrospective study on treatment options and prognosis of patients with advanced-stage CTCL in China showed that patients who received immunomodulatory therapy as first-line treatment had higher response rates and longer survival (35). However, some patients show inadequate response to immunomodulatory therapy and require more intensive targeted therapies or chemotherapy.

By comparing the proteomic profiles of patients who responded to immunomodulatory therapy and those who required targeted therapy or chemotherapy, we identified DEPs involved in intracellular signaling pathways such as RAF/MAP kinase, JAK-STAT, and ERK/MAPK, as well as immune processes like neutrophil degranulation and the complement system. Based on the functional analysis, we propose a multi−regulatory network model associated with treatment resistance: alterations in the function of ECM−related proteins may enhance the migratory and invasive capacity of tumor cells, while changes in the activation levels of intracellular signaling pathways ultimately promote tumor cell proliferation and survival. Together, these mechanisms constitute the molecular basis of treatment resistance in patients.

Among the DEPs, we identified CTSB, GSTO1, and WDFY4 as potential biomarkers to distinguish between the two groups. CTSB, a lysosomal protease, has been shown to be highly expressed in various tumors and plays a role in promoting tumor cell invasion, metastasis, and angiogenesis (36). GSTO1 is a glutathione S-transferase and has been linked to tyrosine kinase inhibitor resistance and disease progression in lung adenocarcinoma (37), consistent with our finding of its upregulation in refractory patients. WDFY4 is involved in antigen cross-presentation, and studies suggest that WDFY4 plays an important role in antitumor immunity (38). In lung adenocarcinoma cell lines, upregulation of WDFY4 induces B cell infiltration and activation, inhibiting tumor growth (39). In our study, we also observed high expression of WDFY4 in the “immunomodulatory therapy group”, suggesting that anti-tumor immunity may correlate with treatment responsiveness in patients.

While the prognosis for advanced-stage CTCL is generally poor, some patients can achieve long-term disease stabilization with appropriate treatment. Previous proteomic studies on early-stage MF patients classified them into aggressive and non-aggressive groups based on overall survival and identified DEPs such as PARP-1, HSAP1L, and HSPA1A, which were upregulated in aggressive MF (40). However, few studies have focused on prognostic biomarkers for advanced CTCL. To investigate proteins and pathways related to disease aggressiveness, we compared the proteomic profiles of patients with and without disease progression during long-term follow-up. The DEPs between these groups were involved in pathways including damage repair, ICOS-ICOSL signaling, IL-4 signaling, and PD-1/PD-L1 signaling. We identified GOLGA1 and STIP1 as potential biomarkers for predicting disease aggressiveness. GOLGA1, a Golgi family protein involved in protein transport, low expression of which has been correlated with poor overall survival in breast cancer (41). STIP1, which coordinates the function of heat shock proteins, has been suggested as a biomarker for ovarian cancer, with higher expression levels correlating with shorter survival and poorer prognosis (42), consistent with our finding that patients with aggressive disease had higher expression of STIP1.

It is important to note that subgroups of patients with different treatment responsiveness and progression risk showed different clinical characteristics, such as stages and lesion types, which are also associated with clinical outcomes. The molecular changes reported here may serve as correlates of these clinical features. Future studies adjusting for clinical covariates are needed to identify the contributions of these molecular signatures.

Several limitations of this study should be acknowledged. First, the small number of patients enrolled and the limited transcriptomic data may affect the results of the correlation analysis between proteomic and transcriptomic data. The results provide a descriptive analysis based on current data, and further validation with a larger sample size is needed. Second, the three molecular subtypes of CTCL were defined and characterized within the discovery dataset, without external validation, due to the lack of publicly available proteomic datasets and an independent cohort. The absence of external validation represents a key limitation. Future work should prioritize testing the reproducibility of the subtype classification in independent datasets. Third, the comparison of p-AKT expression was conducted in seven patients, and the predictive value of p-AKT for the efficacy of PI3K inhibitors wasn’t be validated in an independent cohort. The findings provide preliminary evidence of potential associations, and a formal validation study is needed, ideally with a larger, independent cohort of patients receiving uniform treatment. Additionally, the selected biomarkers were only validated by immunohistochemical staining in small cohorts, and validation for these candidate biomarkers in a larger prospective cohort is needed, and further functional validation studies are needed to explore their clinical application potential.

In summary, our findings point toward the existence of three molecular subtypes of advanced-stage CTCL. The identified subtype-specific proteins and pathways provide a basis for further investigation of therapeutic targets for different subtypes. In addition, our data highlight several candidate biomarkers with potential predictive value for treatment responsiveness and disease aggressiveness. These preliminary findings contribute to the ongoing development of risk-based and personalized treatment strategies for advanced CTCL patients.

## Data Availability

The data presented in the study are deposited in the ProteomeXchange repository, accession number PXD074816 and Genome Sequence Archive repository, accession number HRA016851.
