# Supplementary material for: Proteomics and transcriptomics reveal molecular subtypes and biomarkers of advanced cutaneous T-cell lymphoma
Source: Front Oncol. 2026 Jul 15;16:1849456. doi: 10.3389/fonc.2026.1849456 (PMC13414942; doi:10.3389/fonc.2026.1849456)
Supplement: Supplementary file 8 [file DataSheet1.doc]

**Supplementary figures**


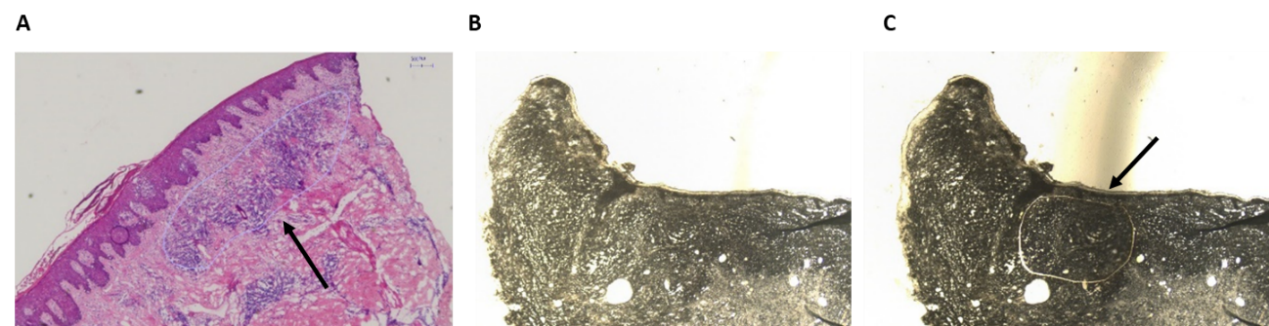


**Figure S1. Laser microdissection workflow.** (A) Tumor cell infiltration zones were delineated on H&E-stained sections (black arrow); (B-C) Corresponding tumor cell-infiltrated areas were selected on frozen sections, with laser capture microdissection performed to isolate tissue sections of selected regions. Representative images showing pre-microdissection (B) and post-microdissection (C) states, the black arrow indicates the cutting area.


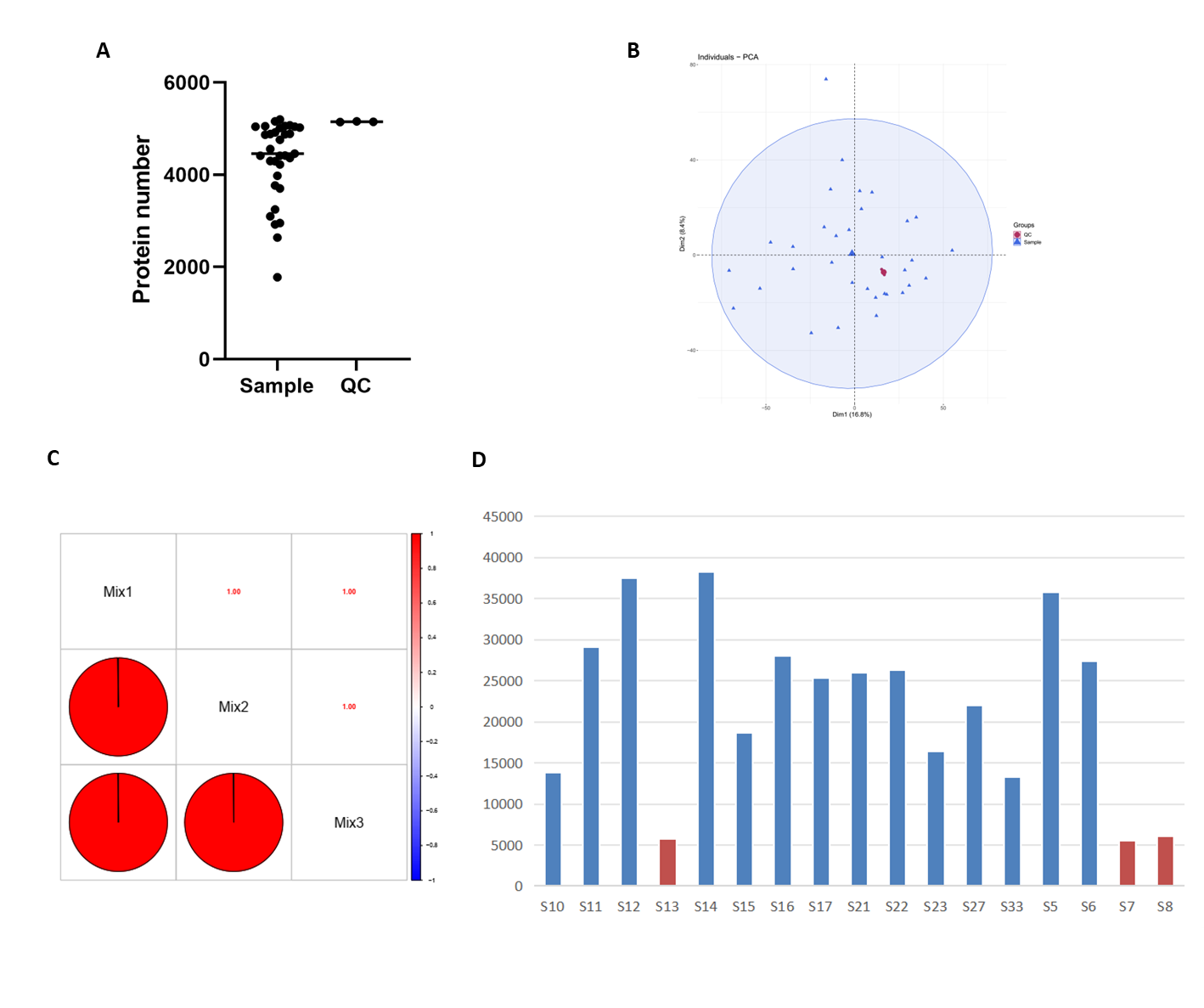


**Figure S2.** **Proteomics Data Quality Control.** (A) The scatter plot of the protein identification number in QCs and Samples. (B) PCA analysis results of the QCs and samples. C: Correlation heatmap of QC samples. D: The Bar plot of the transcript identification number in QCs and Samples. 3 samples (S13, S7, S8) with low transcript identification number and was excluded for further study.

**
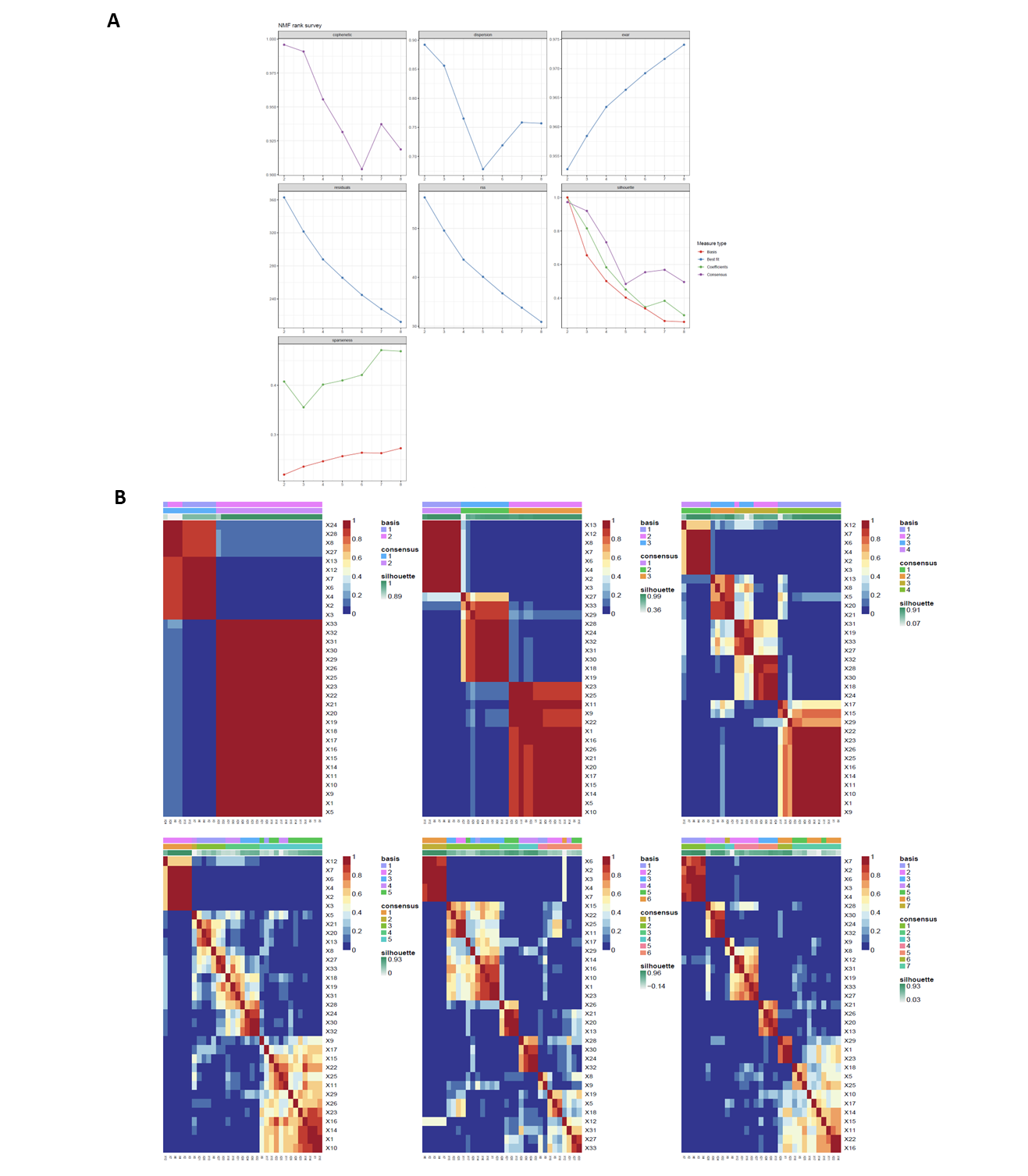
**

**Figure S3.** **Non-negative matrix factorization consensus cluster.** (A) The optimal number of clusters was selected using the cophenetic plot, and silhouette plot. The cophenetic plot showed the best clustering results at k=3. (B) The consensus matrix is derived by consensus clustering, exploring the range of from k=2 to k=7.


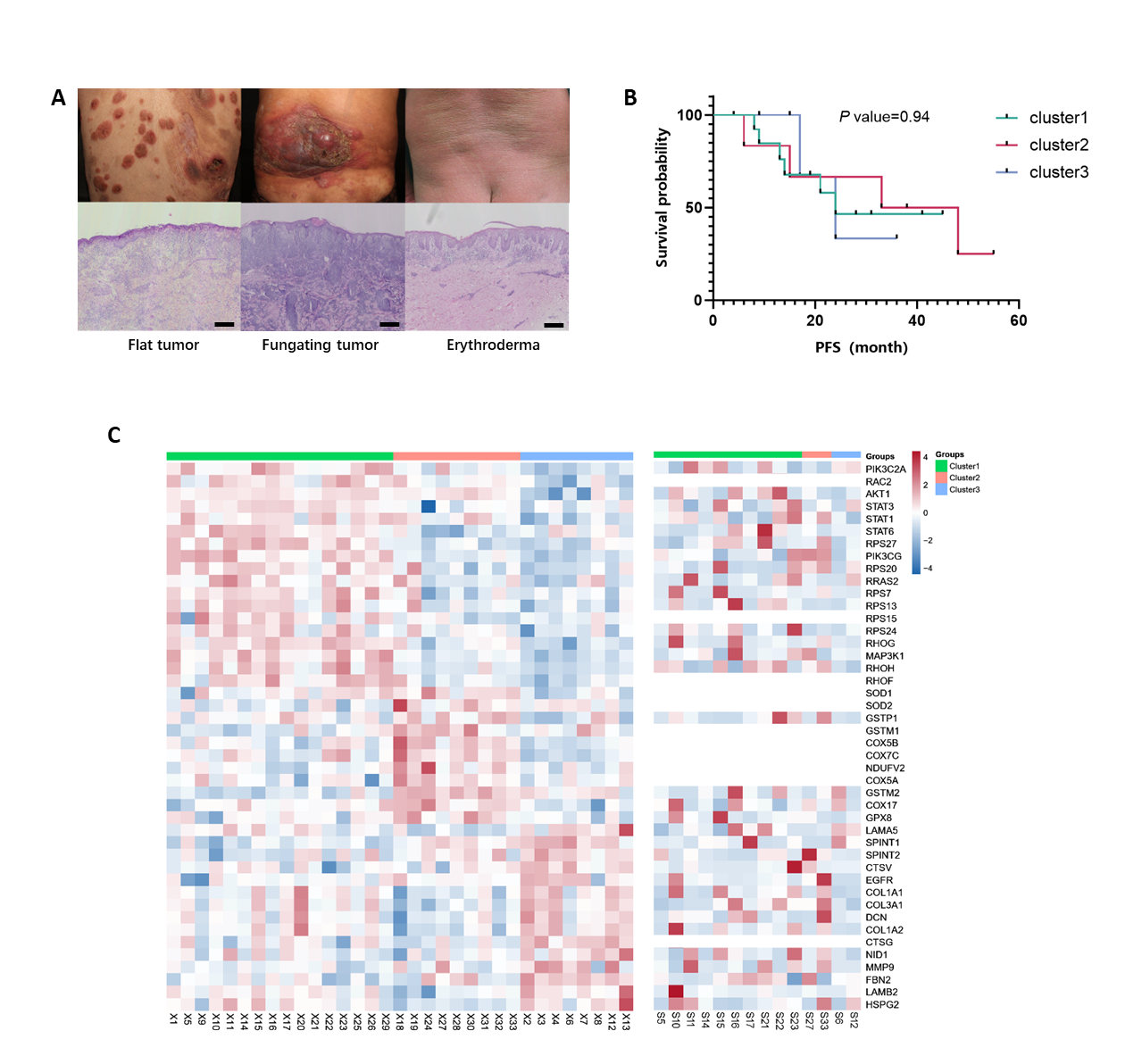


**Figure S4.**  **The clinical manifestations and the prognosis, characteristic protein abundance of the three subtypes.** (A) Clinical and pathological images of the flat tumor-type, fungating tumor-type, and erythrodermic-type (Scale bar=500μm). (B) Kaplan-Meier curves showing the disease progression time for patients with available prognostic information in the three subtypes. (C) Heatmap showing the abundance of proteins involved in the key pathways and corresponding mRNA abundance in the three subtypes.


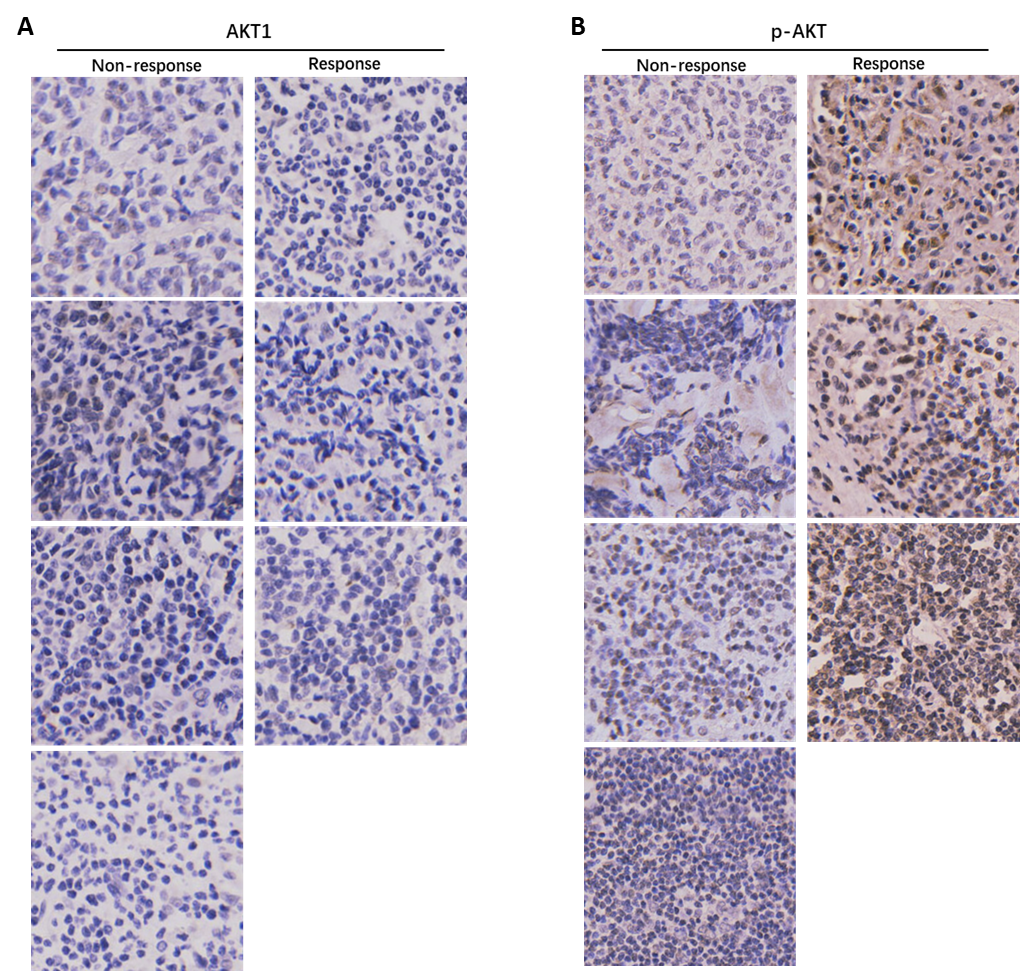


**Figure S5.** **Immunohistochemical results of AKT1 and p-AKT.** Immunohistochemical staining results of AKT1 (A) and p-AKT (B) in the patient cohort receiving PI3K inhibitor treatment.


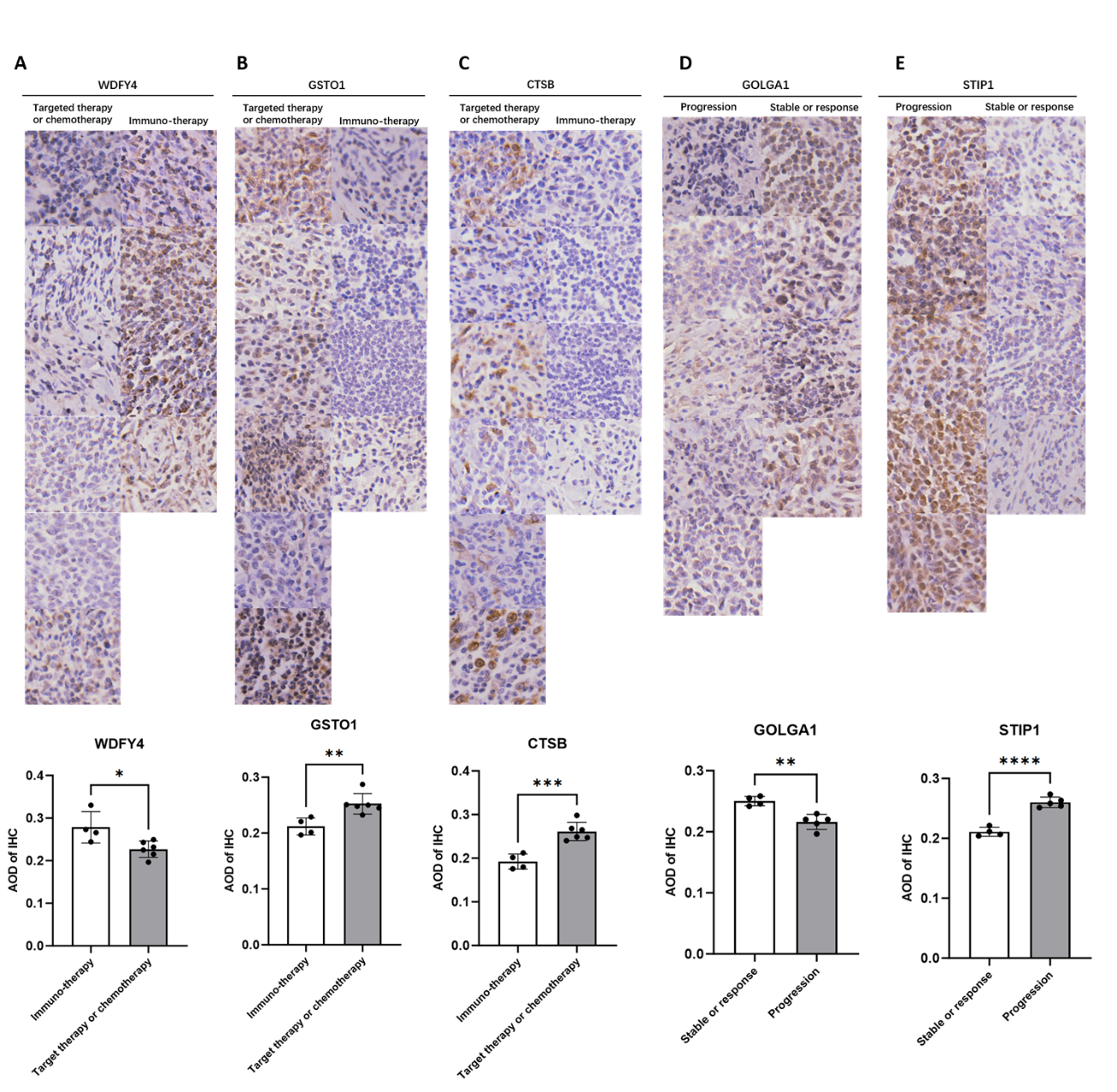


**Figure S6.** **Immunohistochemical results of treatment responsiveness and prognostic biomarkers.** Immunohistochemical staining results of WDFY4 (A), GSTO1 (B), CTSB(C), GOLGA1 (D), STIP1 (E) in the validation patient cohort.
